# Genomewide Association Study for Determinants of HIV-1 Acquisition and Viral Set Point in HIV-1 Serodiscordant Couples with Quantified Virus Exposure

**DOI:** 10.1371/journal.pone.0028632

**Published:** 2011-12-12

**Authors:** Jairam R. Lingappa, Slavé Petrovski, Erin Kahle, Jacques Fellay, Kevin Shianna, M. Juliana McElrath, Katherine K. Thomas, Jared M. Baeten, Connie Celum, Anna Wald, Guy de Bruyn, James I. Mullins, Edith Nakku-Joloba, Carey Farquhar, Max Essex, Deborah Donnell, James Kiarie, Bart Haynes, David Goldstein, for the Partners in Prevention HSV/HIV Transmission Study Team

**Affiliations:** 1 Department of Global Health, University of Washington, Seattle, Washington, United States of America; 2 Department of Medicine, University of Washington, Seattle, Washington, United States of America; 3 Department of Pediatrics, University of Washington, Seattle, Washington, United States of America; 4 Center for Human Genome Variation, School of Medicine, Duke University, Durham, North Carolina, United States of America; 5 The Department of Medicine, Royal Melbourne Hospital, University of Melbourne, Melbourne, Australia; 6 Center for HIV/AIDS Vaccine Immunology, School of Medicine, Duke University, Durham, North Carolina, United States of America; 7 Vaccine and Infectious Diseases Division, Fred Hutchinson Cancer Research Center, Seattle, Washington, United States of America; 8 Department of Epidemiology, University of Washington, Seattle, Washington, United States of America; 9 Perinatal HIV Research Unit, University of the Witwatersrand, Johannesburg, South Africa; 10 Department of Microbiology, University of Washington, Seattle, Washington, United States of America; 11 Mulago Hospital, Makerere University, Kampala, Uganda; 12 Department of Immunology and Infectious Diseases, Harvard University, Boston, Massachusetts, United States of America; 13 Statistical Center for HIV/AIDS, Fred Hutchinson Cancer Research Center, Seattle, Washington, United States of America; 14 Department of Obstetrics and Gynaecology, University of Nairobi, Nairobi, Kenya; University Hospital Zurich, Switzerland

## Abstract

**Background:**

Host genetic factors may be important determinants of HIV-1 sexual acquisition. We performed a genome-wide association study (GWAS) for host genetic variants modifying HIV-1 acquisition and viral control in the context of a cohort of African HIV-1 serodiscordant heterosexual couples. To minimize misclassification of HIV-1 risk, we quantified HIV-1 exposure, using data including plasma HIV-1 concentrations, gender, and condom use.

**Methods:**

We matched couples without HIV-1 seroconversion to those with seroconversion by quantified HIV-1 exposure risk. Logistic regression of single nucleotide polymorphisms (SNPs) for 798 samples from 496 HIV-1 infected and 302 HIV-1 exposed, uninfected individuals was performed to identify factors associated with HIV-1 acquisition. In addition, a linear regression analysis was performed using SNP data from a subset (n = 403) of HIV-1 infected individuals to identify factors predicting plasma HIV-1 concentrations.

**Results:**

After correcting for multiple comparisons, no SNPs were significantly associated with HIV-1 infection status or plasma HIV-1 concentrations.

**Conclusion:**

This GWAS controlling for HIV-1 exposure did not identify common host genotypes influencing HIV-1 acquisition. Alternative strategies, such as large-scale sequencing to identify low frequency variation, should be considered for identifying novel host genetic predictors of HIV-1 acquisition.

## Introduction

HIV-1 interacts with many host factors during the process of infection and replication. However, there is only one confirmed example of a host factor variant that modifies HIV-1 infection outcomes: *CCR5- Δ32*, a variant in the co-receptor for HIV-1 cellular entry has been shown to increase host resistance to HIV-1 infection and HIV-1 disease progression [1]–[3]. While, *CCR5- Δ32* is relatively infrequent or absent in many populations, its existence supports the need to broadly evaluate the human genome for other host genetic variants that influence HIV-1 acquisition.

To date, broad evaluations for host genetic factors have been most successful with identifying predictors of HIV-1 control in infected individuals with recent genome-wide association studies (GWAS) [4]–[6] identifying single nucleotide polymorphisms (SNPs) in the Human Leukocyte Antigen (HLA) complex associated with plasma HIV-1 set point, and disease non-progression [7], as well as genes outside HLA associated with disease progression [8].

Studies looking for host genetic variation associated with HIV-1 acquisition have been more challenging. Candidate variation has been evaluated in HLA, chemokines/chemokine receptors, mediators of the innate, and adaptive immune responses, and factors thought to underlie intracellular viral restriction in diverse epidemiologic contexts (reviewed in reference [9]). To date, no specific gene or variant has been found to consistently influence HIV-1 acquisition/resistance across these diverse studies. Recently, a GWAS evaluated common SNPs across the human genome comparing HIV-1 seropositive to seronegative Malawians and found no common SNPs associated with HIV-1 infection [10].

However, interpretation of studies of HIV-1 acquisition is complicated by the fact that levels of HIV-1 exposure are difficult to quantify, and yet modify risk of HIV-1 sexual transmission by up to 300-fold [11]. Lack of HIV-1 exposure quantification could therefore result in reduced power to detect relevant common variants due to misclassification in assigning HIV-1 acquisition phenotypes (e.g., HIV-1 susceptible individuals with low HIV-1 exposure and therefore at low risk of HIV-1 acquisition who, without quantitative assessment of exposure, are misclassified as HIV-1 resistant).

The principal determinant of HIV-1 sexual transmission risk, and therefore primary HIV-1 exposure factor, is the plasma HIV-1 RNA level in the transmitting partner [12], [13]. Other epidemiologic, biologic and behavioral factors (e.g., circumcision status of male uninfected partners, and frequency of unprotected sex between partners) also contribute to this risk [11], [14]–[16]. Thus, accurate quantification of the level of HIV-1 exposure and associated HIV-1 sexual transmission risk requires data from both sexual partners.

Studies of HIV-1 serodiscordant couples (one partner HIV-1 infected and the other HIV-1 uninfected) offer unique advantages for identifying factors associated with HIV-1 acquisition. In particular, prospective collection of specimens and data from both sexual partners facilitates quantification of HIV-1 exposure risk, and confirmation of HIV-1 transmission linkage between partners. Therefore, this study design allows HIV-1 uninfected individuals with little to no HIV-1 exposure to be excluded from the analysis. To date, no GWAS for host genetic factors underlying HIV-1 acquisition has been performed in a cohort of HIV-1 serodiscordant couples. Here we report use of specimens and data from African heterosexual HIV-1 serodiscordant couples in a GWAS for host genetic predictors of HIV-1 acquisition and set point plasma RNA levels.

## Methods

### Study Cohort

Study participants were selected from two cohorts of African HIV-1 serodiscordant heterosexual couples:

1. The Partners in Prevention HSV/HIV Transmission Study enrolled 3408 African HIV-1 serodiscordant couples at 14 sites in East and Southern Africa, and followed them quarterly for up to 24 months to evaluate the efficacy of herpes simplex virus type-2 (HSV-2) suppression to reduce HIV-1 transmission to their heterosexual HIV-1 uninfected partners [17], [18]. HIV-1 infected partners in this trial were required to be dually-infected with HSV-2 with a CD4 count ≥250 cells/mm^3^; there was no eligibility criterion related to HSV-2 serostatus of the HIV-1 uninfected partner. The primary analysis for this trial found acyclovir suppression reduced plasma HIV-1 level of the HIV-1 infected partners by a mean of 0.25 log_10_ copies/ml, but did not reduce the risk of HIV-1 transmission [18].
2. The Couples Observational Study (COS) used a similar recruitment process to enroll 485 HIV-1 serodiscordant couples from Soweto, South Africa and Kampala, Uganda without restriction on CD4 count or HSV-2 serostatus of the HIV-1 infected partner; these couples were followed quarterly for up to 12 months.

In both cohorts, HIV-1 serostatus in the initially HIV-1 uninfected partner was assessed by dual HIV-1 rapid assays and HIV-1 seroconversions confirmed by ELISA, and Western blot or RT-PCR [18]. Plasma HIV-1 *env* and *gag* sequencing of both partners were compared with those consistent with transmission linkage within the partnership classified as “linked” [18], [19]. Seroconverting partners were also followed after seroconversion to document plasma HIV-1 RNA set point and CD4 counts.

Among all participants recruited at both COS study sites and at the 10 Partners in Prevention HSV/HIV Transmission Study sites at which consent for host genetic studies had been obtained, a total of 863 individuals were selected for genotyping. Procedures used to identify these individuals are described below.

### Sample selection

In order to identify HIV-1 non-seroconverting and seroconverting partners with similar ranges of HIV-1 exposure we identified epidemiologic factors predicting HIV-1 transmission. Baseline data from linked transmitting and non-transmitting couples identified in the Partners in Prevention HSV/HIV Transmission Study was used to develop a Cox proportional hazards model identifying HIV-1 exposure factors associated with HIV-1 transmission: gender, age, male circumcision, HIV-1 infected partner plasma RNA level, and unprotected sex. Seroconverting couples in either cohort were matched to two non-seroconverting couples based on baseline status for each HIV-1 exposure factor. To augment power to detect genotypes associated with host resistance to HIV-1 we also included additional HIV-1 uninfected individuals with all HIV-1 exposure factors in high-risk strata.

To facilitate comparisons of HIV-1 exposure levels we used the regression coefficients of the Cox prediction model to develop an exposure score that ranged from 0 (lowest exposure) to 7 (highest exposure) (Table 1). The cumulative risk of HIV-1 infection for individuals with an exposure risk score ≥2 was 31-fold greater than for those with an exposure risk <2 (4.99% vs 0.16%).

**Table 1:** Identification of HIV-1 exposure factors through a predictive model of HIV-1 transmission.

Parameter	P-value(linked infections)	Parameter estimate	Hazard Ratio	Exposure Score
Any unprotected sex	<0.001	0.60	1.82	1
Male uninfected partner uncircumcised	0.028	0.59	1.81	1
Uninfected partner age<25 yrs	0.022	0.56	1.74	1
Infected partner plasma viral RNA (<10,000 copies/ml – baseline)				
10–50,000 copies/ml	<0.001	1.32	3.75	2
50–100,000 copies/ml	<0.001	2.25	9.46	4
>100,000 copies/ml	<0.001	2.00	7.38	4

Baseline data from non-transmitting and linked transmitting couples from the Partners in Prevention HSV/HIV Transmission Study (N = 3360) was used to develop a predictive Cox regression model of HIV-1 transmission. An exposure risk score based on model regression coefficients was developed to quantify exposure risk and confirm that participants selected for GWAS testing who had not seroconverted did have HIV-1 exposure.

Among the 863 individuals identified for genotyping through this process, 512 (59%) were HIV-1 infected (384 [75%] prevalent and 127 [25%] incident HIV-1 infections) and 352 (41%) remained HIV-1 uninfected despite documented HIV-1 exposure. Table 2 provides a breakdown of the sample selection by HIV-1 infected and uninfected status.

**Table 2:** Summary of Sample Selection for Genotyping.

	Total couples genotyped(% Exposure with score ≥5)	Total individuals(% East Africa)	Prevalent HIV-1+(% East Africa)	Seroconverters(% East Africa)	HIV-1 Uninfected(% East Africa)
Participants from couples associated with seroconversion	127 (50)	254 (74.8%)	127 (74.8%)	127 (74.8%)	–
Participants fromnon-seroconverting couples	257 (43)	514 (75.9%)	257 (75.9%)	–	257 (75.9%)
UnmatchedHIV-1 uninfected individuals	–	95 (82%)	0	0	95 (82%)
Total individuals for genotyping		863 (76.2%)	384 (75.5%)	127 (74.8%)	352 (77.6%)
Excluded Individuals					
Failed genotyping		12	3	1	8
Gender mismatch		11	5	1	5
Cryptic relatedness		2	0	1	1
Exposure score<2		32	–	–	32
Population outlier		8	2	2	4
Total participants included inHIV-1 acquisition analysis		798 (76.1%)	374 (75.7%)	122 (75.4%)	302 (76.8%)
Total participants included inHIV-1 set point analysis		403 (74.7%)	293 (74.4%)	110 (75.5%)	–

### Genotyping

Genomic DNA was extracted from 1 ml archived whole blood. All samples were genotyped using Illumina HumanHap 1M-Duo (*n*p135) Bead Chips [20], which feature more than 1 million SNPs including 21 directly genotyped variants that have been identified in previous studies as associated with HIV-1 susceptibility [4]. SNPs with a call frequency of <99%, with minor allele frequency <1% or with >5% missing results were excluded leaving 990,115 SNPs for association analysis. Bonferroni correction for multiple testing used a *P* value cutoff of 5.1×10^−8^ for genome-wide significance.

### Candidate SNP subset

As a subanalysis, we evaluated 21 candidate SNPs previously implicated in HIV-1 infection that were present on the 1M-Duo chip platform. We report uncorrected p-values for these 21 SNPs.

### Statistical analysis

#### Sample exclusions

A total of 25 samples were excluded based on genotyping quality control steps (twelve samples failed genotyping, eleven had genotype inconsistent with epidemiologically assigned gender, and two failed cryptic relatedness requirements). Population structure was evaluated using a modified EIGENSTRAT method [21], the first principal component (eigenvector) discriminated individuals based on whether they were from Southern African (South Africa and Botswana) or Eastern African (Kenya, Uganda and Tanzania) study sites (Figure S1); at this step, eight samples were removed as population outliers.

Finally, in order to capture all HIV-1 infected individuals for genotyping, the initial matching for HIV-1 exposure included all seroconverting couples. Thus, some HIV-1 uninfected partners were selected for genotyping by matching to HIV-1 exposure scores of unlinked seroconverting couples; many of these HIV-1 uninfected partners had HIV-1 exposure risk scores<2. However, since all couples with linked transmission had HIV-1 exposure scores ≥2, we took this as an HIV-1 exposure cutoff and excluded from analysis 32 HIV-1 uninfected individuals with exposure score <2.

#### Analysis of specific HIV-1 phenotypes

1) HIV-1 susceptibility analysis: We evaluated genotypes for all HIV-1 seropositive individuals, including prevalent HIV-1 infections (partners who were HIV-1 infected at enrollment), and incident infections (partners HIV-1 seronegative at enrollment who became infected during follow-up). These were compared to genotypes for all HIV-1 exposed, seronegative individuals (HIV-1 exposures scores ≥2). We also compared genotypes of HIV-1 seropositive individuals to the subset of all HIV-1 uninfected individuals with all baseline HIV-1 exposure characteristics in the highest risk strata. For both of these analyses, we performed standard logistic regression, additive genetic model, in PLINK (version 1.07) [22], [23], using gender, age and the individual coordinates of six EIGENSTRAT eigenvectors as covariates.

2) Plasma RNA set point analysis: Similar to previous analyses [24] we defined the plasma HIV-1 RNA set point among individuals with prevalent infection (partner who was HIV-1 infected at enrollment) as the average log_10_ plasma RNA level after excluding RNA measurements taken at or after the initiation of antiretroviral therapy (ART) or when CD4 count was <200 cells/mm^3^. We required RNA measurements to be stable, with measurements for each individual visually inspected for notable discrepancies (e.g., no plasma HIV-1 RNA measurements from each individual differing by >1 log copies/ml); we required a minimum of 2 reliable and consistent measurements per individual. For individuals with incident infection (e.g., HIV-1 seroconversion during follow-up) an estimated date of HIV-1 infection was established based on a combination of HIV-1 serology and plasma HIV-1 RNA PCR results, with HIV-1 set point calculated as the average of all log_10_ plasma HIV-1 RNA measurements taken 4 months or more after the estimated date of infection [25]. For all analyses, plasma HIV-1 RNA levels below the limit of detection (240 copies/mL) were set to 120 copies/mL. For this analysis, we performed linear regression for set point using age, gender, acyclovir randomization, seroprevalent vs. seroconverter status and five EIGENSTRAT eigenvectors as covariates.

### Ethical Review

All individuals whose samples were evaluated through this genotyping provided informed consent for storage of samples for future research including genetic studies. Human subject review and approval for this analysis was obtained at the University of Washington and at local and affiliated institutional review boards for study sites where participants were enrolled. The Partners in Prevention HSV/HIV Transmission Study was registered with ClinicalTrials.gov (#NCT00194519).

## Results

### Genome-wide common variation associated with HIV-1 acquisition

After quality control, 798 samples remained for analysis (496 from HIV-1 infected and 302 from HIV-1 uninfected individuals). Table 3 shows epidemiological and clinical characteristics of these individuals.

**Table 3:** Epidemiologic Characteristics of Individuals in HIV-1 Acquisition Analysis.

*HIV-1 Infected Partners*			
Characteristic	PrevalentHIV-1 infected	Incident HIV-1 infected	All
Number	374	122	496
% Female	205 (55%)	55 (45%)	260 (52%)
% recruited from East African sites	283 (76%)	92 (75%)	375 (76%)
Median age [range] (years)	32 [18–67]	30 [18–72]	31 [18–72]
Baseline plasma HIV-1 RNA level or plasma HIV-1 set point (median log_10_ copies/ml)	4.62	4.49	n/a
Baseline plasma HIV-1 RNA level ofHIV-1 infected partner (median log_10_ copies/ml)	n/a	4.26	n/a
Median CD4 count at enrollment (cells/ul)	413	n/a	n/a
Transmission linkage confirmed*	n/a	86 (70%)	n/a

*HIV-1 Uninfected Partners*			
Characteristic	MatchedHIV-1 uninfected	Additional unmatchedHIV-1 uninfected	All
Number	223	79	302
% Female	110 (49%)	41 (52%)	151 (50%)
% recruited from East African sites	168 (75%)	64 (81%)	375 (77%)
Median age [range] (years)	31 [18–70]	28 [19–66]	30.5 [18–70]
Baseline HIV-1 exposure characteristics			
Highly exposed (risk score ≥5)	13 (5.8%)	77 (97.5%)	90 (29.8%)
Baseline plasma HIV-1 RNA of			
HIV-1 infected partner (median log_10_ copies/ml)	4.52	5.14	4.83
% male uncircumcised	62.8%	76.3%	66.2%
% age <25 years	65%	76%	67.9%
% reporting unprotected sex	24.7%	95%	43%

*Based on plasma virus sequencing HIV-1 *env* and *gag* of both transmitting and seroconverting partner-pairs.

**Figure 1:**
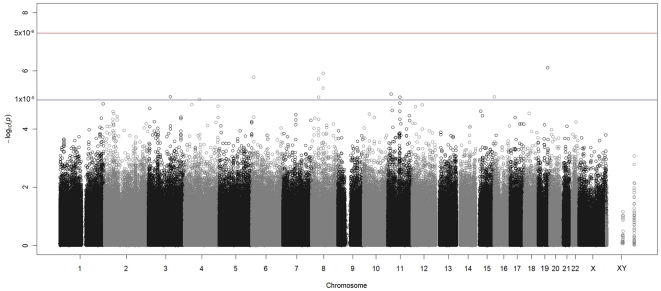
Manhattan plot for analysis of HIV-1 acquisition. -log_10_(p) is plotted for all SNPs against physical location of each SNP in the genome (listed by chromosome number 1 through 22, and X and XY). The threshold for genome-wide significance (P = 5.1×10^−8^) is indicated.

In the multivariate regression analysis, no single SNP reached genome-wide significance of *p*<5.1×10^−8^ (Figure 1). Furthermore, a meta-analysis based on the two separate analyses for Eastern and Southern African recruited individuals was consistent with the results from the pooled analysis. An annotated list of all SNPs with *p*<1×10^−5^ based on the pooled analysis of Eastern and Southern recruited Africans is provided (Table S1). Among the 21 SNPs available on the 1M-Duo chip platform that have previously been implicated with HIV-1 acquisition, none were GWAS significant; only two were significant at a *p*<0.05 threshold with both of these having effects in the opposite direction from the original findings: for rs2070729-*IRF1* the G allele was linked to increased susceptibility *(p* = 0.01) in contrast to the original study, [26], and for rs1800451-*MBL2* the A allele was linked to reduced susceptibility (*p* = 0.02) in contrast to prior studies [27]–[32] (Table S2).

Comparison of the 496 HIV-1 infected individuals to 90 HIV-1 uninfected individuals having the higher HIV-1 exposure risk scores (>5 risk score) did not identify SNPs reaching genome-wide significance.

### Genome-wide common variation associated with HIV-1 set point

**Figure 2:**
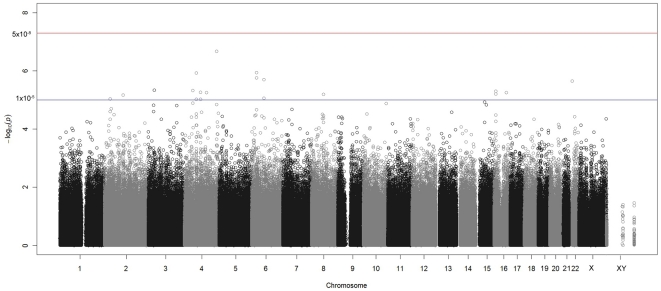
Manhattan plot for analysis of plasma HIV-1 set point. -log_10_(p) is plotted for all SNPs against physical location of each SNP in the genome (listed by chromosome number 1 through 22, and X and XY). The threshold for genome-wide significance is indicated.

Among the 496 HIV-1 infected individuals in our cohort, 403 (81%) met the requirements for stable HIV-1 set point including 293 (73%) prevalent and 110 (27%) incident infections (Table 4). Comparison of log_10_ plasma HIV-1 levels of prevalent and incident infections showed no statistically significant difference between them (*p* = 0.14), so both groups were combined for subsequent analyses. The overall median plasma HIV-1 level for individuals included in this plasma HIV-1 set point analysis was 4.53 log_10_ copies/ml. The median plasma HIV-1 level of males (n = 180) was 4.57 log_10_ copies/ml compared to females (n = 223) 4.51 log_10_ copies/ml (*p* = 0.57). Linear regression performed on these 403 HIV-1 infected individuals for the 990,115 SNPs that passed quality control found no single SNP reaching genome-wide significance (Figure 2). An annotated list of all markers obtaining a *P*-value less than 1×10^−5^ was generated using WGAviewer software [16] (Table S3).

**Table 4:** Epidemiologic Characteristics of HIV-1 Infected Partners in HIV-1 Set Point Analysis.

Characteristic of HIV-1 Infected Participants for HIV-1 Set Point Analysis	Prevalent HIV-1 Infected	Incident HIV-1 infected	All
Number	293	110	403
% Female	176 (60%)	47 (42.7%)	223 (55.3%)
% recruited from East African sites	218 (74%)	83 (75%)	301 (75%)
Median age [range] (years)	31 [18–67]	30 [18–54]	31 [18–67]
Baseline plasma HIV-1 RNA or plasma HIV-1 set point (median log_10_ c/ml)	4.57	4.49	4.53
Baseline plasma HIV-1 RNA level of HIV-1 infected partner (median log_10_ c/ml)	n/a	4.17	n/a
Median CD4 count at enrollment (cells/ul)	436	n/a	n/a

## Discussion

We found no common SNPs associated with HIV-1 acquisition at genome-wide significance. This result is consistent with a recent GWAS of Africans recruited from a high-risk setting [10]. Our study is the first to select participants based on HIV-1 exposure levels ensuring that HIV-1 uninfected individuals had documented risk for HIV-1 acquisition. Furthermore, we also found that, among the subset of African HIV-1 infected participants with stable plasma HIV-1 level (combining individuals with incident and chronic HIV-1 infection), no SNPs on the 1M-Duo chip were associated at genome-wide significance with plasma HIV-1 set point. This is also similar to recent findings from a GWAS for determinants of plasma HIV-1 set point in an African-American cohort [6].

Three important limitations to this analysis must be considered in interpreting our findings. First, our overall sample size (n = 798 individuals passing quality control criteria) had sufficient power to detect host genetic variants with large effect sizes: e.g., variants with minor allele frequencies of 5% or 20% having a genotype relative risk greater than 3.2 and 2.1, respectively. A larger cohort would be needed to evaluate for host genetic factors associated with smaller genotype relative risks. However, it is possible that lower levels of linkage disequilibrium, particularly within the MHC region in African populations, reduced our ability to identify MHC genetic variants potentially associated to plasma HIV-1 set point in this cohort. This was apparent in a previous analysis for host genetic determinants of set point viral load in an African-American cohort [6]. Although two of the top four SNPs (rs10484434 and rs11755492 –Table S3) in our analysis for determinants of set point are located physically close to the MHC region, there is no evidence that these SNPs tag causative variants within the MHC for our cohort. Additional studies and meta-analyses of these SNPs may provide further information on whether these SNPs may have a weak association with set point that we were not powered to detect. Finally, in addition to reducing our power to detect host genetic association with set point, the lower levels of linkage disequilibrium in African populations might have reduced our power to detect weaker host genetic associations with HIV-1 acquisition.

Second, the set of common variants evaluated for association with HIV-1 outcomes is based on HapMap data derived from common variation in West African (Yoruban) populations and likely does not capture detailed host variation across diverse subSaharan African populations [33]–[35]. Our analysis of population structure did provide clear discrimination of persons of Southern African and Eastern African origin (Figure S1a and S1b), However, improved databases of common host genetic variation in East and Southern African populations are becoming available through the recently completed 1000 genomes project [36]. Nevertheless, the capacity to indirectly capture overall host variation through linkage disequilibrium will still be lower in African populations due to lower levels of linkage disequilibrium present in African populations [35], [37].

Finally, recent studies suggest that low frequency or rare host variation that cannot be readily captured through GWAS analysis is an important source of factors contributing to human disease causation [38], [39]. Such causal rare variation can only be captured through large-scale genome sequencing efforts.

A unique component to our analysis was our use of clinical and behavioral factors from HIV-1 serodiscordant couples to quantify overall HIV-1 exposure risk. This study design provides unique advantages for controlling for epidemiological modifiers of HIV-1 acquisition – both explicitly for factors that are known to influence HIV-1 acquisition (which we have done through the exposure matching), and implicitly for any unidentified exposure factors shared within the partnership. While consanguinity of partners is a potential source of bias in this approach, across all samples, our analysis found only 2 pairs of samples with cryptic relatedness, and one sample from each cryptically related pair was excluded from the analysis. Consistent with previous epidemiologic analyses of HIV-1 transmission risk performed in this and other cohorts [12], [13], [15], [40], plasma HIV-1 RNA in the HIV-1 infected partner has the greatest impact on estimated HIV-1 exposure level. Although we also included additional factors from the HIV-1 uninfected partner (e.g., history of any unprotected sex, circumcision status of male HIV-1 uninfected partners, and age<35 years for HIV-1 uninfected female partners) in our exposure risk quantification, these factors contribute to a much smaller degree to HIV-1 transmission risk. We also did not adjust our findings for the direction of transmission (male-to-female versus female-to-male) since a recent per contact analysis for HIV-1 transmission risk in this cohort found that, after adjusting for plasma HIV-1 levels, the relative risk for male-to-female versus female-to-male transmission was 1.03 (p = 0.93) [41]. Our HIV-1 exposure risk score correlated well with overall proportion acquiring HIV-1 infection, with those having exposure scores ≥2 having a 31-fold increased risk of HIV-1 infection. However, our overall analysis included individuals with a range of HIV-1 exposure levels with limited power to evaluate only those HIV-1 uninfected individuals at the highest HIV-1 exposure levels. Finally, we also did not account for longitudinal changes in exposure risk (e.g., HIV-1 infected partners initiating antiretroviral therapy resulting in reduced plasma HIV-1 levels, or behavioral changes related to frequency of sex or use of condoms). Thus, it remains an open question whether the search for genomic factors underlying HIV-1 acquisition might benefit from identifying individuals with extreme transmission phenotypes, e.g., those who remain HIV-1 seronegative despite persistently high HIV-1 exposures.

In summary, our GWAS comparing HIV-1 infected individuals to HIV-1 uninfected individuals with documented HIV-1 exposure risk did not identify host genetic factors strongly modifying risk of HIV-1 acquisition. Future studies of HIV-1 acquisition and set point determination may benefit from use of larger sample sizes, identification of extreme transmission phenotypes, and large-scale sequencing technologies to capture rare and previously uncharacterized common variants in these African cohorts.

## Supporting Information

Figure S1 **Plot of PC1 versus PC2 population substructure after removal of outliers.** After removing the eight outlier samples, EIGENSTRAT was re-run to obtain the eigenvectors for use as covariates in association analysis. Graphical plots are by A) Region, with black indicating individuals recruited from study sites in Southern African countries (South Africa and Botswana), and red indicating individuals recruited from study sites in East African countries (Kenya, Uganda and Tanzania), and B) HIV-1 status, with black indicating individuals who remained HIV-1 seronegative, and red indicating HIV-1 seropositive partners and individuals who seroconverted.
(DOC)

Table S1 **SNPs associated with p<10^−5^ for HIV-1 susceptibility/resistance.** SNP rs identifier, uncorrected p-value, Chromosome number and basepair position (build 36.3, hg18), a description of the relative position of the SNP in the closest gene, Minor Allele Frequency (MAF) in HIV-1-negative and HIV-1-positive populations, and name and distance to closest gene are as indicated.
(DOC)

Table S2 **List of tested variants previously reported to have an association with HIV-1 susceptibility/resistance.** SNPs listed are those present on the Illumina HumanHap 1M-Duo (*n*p135) Bead Chips that have been previously implicated in candidate gene studies as having impact on HIV-1 acquisition. Characteristics of the studies that reported those previous associations are described.
(DOC)

Table S3 **Variants with p<10^−5^ in HIV-1 set point analysis.** SNP rs identifier, uncorrected p-value, chromosome number and basepair position (build 36.3, hg18), and name and distance to closest gene are as indicated.
(DOC)
