# Correction: Genomewide Association Study for Determinants of HIV-1 Acquisition and Viral Set Point in HIV-1 Serodiscordant Couples with Quantified Virus Exposure

**DOI:** 10.1371/annotation/1dcd8ee4-bcf4-4cf6-97a3-2e26f0f77060

**Published:** 2012-01-17

**Authors:** Jairam R. Lingappa, Slavé Petrovski, Erin Kahle, Jacques Fellay, Kevin Shianna, M. Juliana McElrath, Katherine K. Thomas, Jared M. Baeten, Connie Celum, Anna Wald, Guy de Bruyn, James I. Mullins, Edith Nakku-Joloba, Carey Farquhar, Max Essex, Deborah Donnell, James Kiarie, Bart Haynes, David Goldstein, for the Partners in Prevention HSV/HIV Transmission Study Team

There was an error in the names of the 5th and 19th authors. The correct name of the 5th author is Kevin V. Shianna. The correct name of the 19th author is David B. Goldstein. The corrected citation is: Lingappa JR, Petrovski S, Kahle E, Fellay J, Shianna KV, et al. (2011) Genomewide Association Study for Determinants of HIV-1 Acquisition and Viral Set Point in HIV-1 Serodiscordant Couples with Quantified Virus Exposure. PLoS ONE 6(12): e28632. doi:10.1371/journal.pone.0028632. The corrrected author contributions is: Conceived and designed the experiments: JRL SP JF MJM JMB CC AW JIM DD BH DBG. Performed the experiments: JRL SP KVS CC AW GdB JIM EN-J CF ME JK DBG. Analyzed the data: JRL SP EK KKT. Wrote the paper: JRL SP EK JF KS MJM KKT JMB CC AW GdB JIM EN-J CF ME DD JK DBG.

