# Supplementary material for: Genomewide Association Study for Determinants of HIV-1 Acquisition and Viral Set Point in HIV-1 Serodiscordant Couples with Quantified Virus Exposure
Source: PLoS One. 2011 Dec 12;6(12):e28632. doi: 10.1371/journal.pone.0028632 (PMC3236203; doi:10.1371/journal.pone.0028632)
Supplement: Figure S1 — Plot of PC1 versus PC2 population substructure after removal of outliers. After removing the eight outlier samples, EIGENSTRAT was re-run to obtain the eigenvectors for use as covariates in association analysis. Graphical plots are by A) Region, with black indicating individuals recruited from study sites in Southern African countries (South Africa and Botswana), and red indicating individuals recruited from study sites in East African countries (Kenya, Uganda and Tanzania), and B) HIV-1 status, with black indicating individuals who remained HIV-1 seronegative, and red indicating HIV-1 seropositive partners and individuals who seroconverted. (DOC) [file pone.0028632.s001.doc]

**Figure S1**

Black = individuals recruited from study sites in Southern African countries (South Africa and Botswana), and Red = individuals recruited from study sites in East African countries (Kenya, Uganda and Tanzania).

**Figure 1b:** Black = individuals who remained HIV-1 seronegative, and Red = HIV-1 seropositive partners and individuals who seroconverted.

**A.**

**B.**
