# Supplementary material for: Genomewide Association Study for Determinants of HIV-1 Acquisition and Viral Set Point in HIV-1 Serodiscordant Couples with Quantified Virus Exposure
Source: PLoS One. 2011 Dec 12;6(12):e28632. doi: 10.1371/journal.pone.0028632 (PMC3236203; doi:10.1371/journal.pone.0028632)
Supplement: Table S1 — SNPs associated with p<10−5 for HIV-1 susceptibility/resistance. SNP rs identifier, uncorrected p-value, Chromosome number and basepair position (build 36.3, hg18), a description of the relative position of the SNP in the closest gene, Minor Allele Frequency (MAF) in HIV-1-negative and HIV-1-positive populations, and name and distance to closest gene are as indicated. (DOC) [file pone.0028632.s002.doc]

**Table S1**

| **SNP** | **P** | **Chr** | **Coordinate** | **SNP type** | **MAF**  **(HIV-1 negative)** | **MAF**  **(HIV-1 positive)** | **Closest gene** | **Distance to gene** |
| --- | --- | --- | --- | --- | --- | --- | --- | --- |
| rs3745760 | 7.83X10^-07^ | 19 | 52897537 | 3PRIME_UTR | 0.31 | 0.44 | GLTSCR1 | 0 |
| rs10808739 | 1.21X10^-06^ | 8 | 65802814 | INTRONIC | 0.25 | 0.37 | CYP7B1 | 0 |
| rs4437462 | 1.68X10^-06^ | 6 | 11418405 | INTERGENIC | 0.24 | 0.15 | NEDD9 | -76728 |
| rs1349547 | 1.88X10^-06^ | 8 | 39667535 | INTRONIC | 0.38 | 0.27 | ADAM18 | 0 |
| rs10957322 | 3.90X10^-06^ | 8 | 65832918 | INTRONIC | 0.24 | 0.36 | CYP7B1 | 0 |
| rs10741780 | 6.24X10^-06^ | 11 | 19556547 | INTERGENIC | 0.33 | 0.45 | AC023950.6 | 12732 |
| rs796825 | 7.81X10^-06^ | 3 | 121500170 | INTERGENIC | 0.17 | 0.27 | LRRC58 | 26096 |
| rs4349147 | 7.91X10^-06^ | 16 | 3064921 | INTERGENIC | 0.31 | 0.20 | IL32 | 5252 |
| rs7122539 | 8.00X10^-06^ | 11 | 66419307 | INTRONIC | 0.32 | 0.42 | PC | 0 |
| rs3735880 | 8.03X10^-06^ | 8 | 39624983 | INTRONIC | 0.50 | 0.40 | ADAM18 | 0 |
| rs3796804 | 9.47X10^-06^ | 4 | 82228731 | DOWNSTREAM | 0.26 | 0.17 | PRKG2 | 130 |
| rs35419027 | 1.28X10^-05^ | 11 | 66417525 | INTRONIC | 0.34 | 0.44 | N/A | -9 |
| rs7528333 | 1.38X10^-05^ | 1 | 240305374 | INTERGENIC | 0.11 | 0.20 | PLD5 | 12938 |
| rs4560447 | 1.45X10^-05^ | 4 | 41142267 | INTRONIC | 0.52 | 0.40 | LIMCH1 | 0 |
| rs2069501 | 1.48X10^-05^ | 12 | 56431048 | SYN_CODING | 0.07 | 0.03 | CDK4 | 0 |
| rs10023344 | 1.63X10^-05^ | 4 | 186236342 | INTERGENIC | 0.06 | 0.13 | AC093824.3-2 | 33023 |
| rs7131725 | 1.72X10^-05^ | 12 | 19719923 | INTERGENIC | 0.17 | 0.10 | AC090059.9 | -48713 |
| rs13326304 | 1.95X10^-05^ | 3 | 6650541 | INTERGENIC | 0.24 | 0.16 | GRM7 | -227534 |
| rs4317983 | 2.28X10^-05^ | 11 | 23734035 | INTERGENIC | 0.41 | 0.30 | AC099842.2-1 | -33385 |
| rs7948839 | 2.44X10^-05^ | 11 | 66390973 | INTRONIC | 0.35 | 0.45 | LRFN4 | 0 |
| rs2636061 | 2.45X10^-05^ | 15 | 27212047 | INTRONIC | 0.29 | 0.20 | AC024474.8 | 0 |
| rs11694783 | 2.49X10^-05^ | 2 | 47680185 | INTERGENIC | 0.56 | 0.46 | KCNK12 | -29183 |
| rs1344050 | 2.87X10^-05^ | 18 | 26177559 | INTERGENIC | 0.59 | 0.49 | N/A | -9 |
| rs11889551 | 2.96X10^-05^ | 2 | 50056457 | INTRONIC | 0.47 | 0.37 | NRXN1 | 0 |
| rs11812929 | 3.03X10^-05^ | 10 | 33677249 | INTERGENIC | 0.20 | 0.12 | NRP1 | -12053 |
| rs11769825 | 3.21X10^-05^ | 7 | 72739073 | INTRONIC | 0.05 | 0.11 | WBSCR22 | 0 |
| rs7949316 | 3.50X10^-05^ | 11 | 120000414 | INTERGENIC | 0.18 | 0.28 | GRIK4 | -35767 |
| rs11639006 | 3.51X10^-05^ | 15 | 37100474 | INTERGENIC | 0.47 | 0.37 | AC087878.7 | 5100 |
| rs7568216 | 3.66X10^-05^ | 2 | 70269701 | INTRONIC | 0.19 | 0.28 | C2orf42 | 0 |
| rs6786409 | 3.90X10^-05^ | 3 | 180528187 | INTRONIC | 0.26 | 0.17 | ZNF639 | 0 |
| rs2327459 | 3.93X10^-05^ | 6 | 11808424 | INTERGENIC | 0.14 | 0.22 | AL022724.2 | -9614 |
| rs3775543 | 4.00X10^-05^ | 4 | 185547212 | SPLICE_SITE,SYN_CODING | 0.35 | 0.26 | IRF2 | 0 |
| rs8076276 | 4.01X10^-05^ | 17 | 27527068 | INTRONIC | 0.24 | 0.16 | RHOT1 | 0 |
| rs10994023 | 4.05X10^-05^ | 10 | 61232983 | INTRONIC | 0.27 | 0.18 | CCDC6 | 0 |
| rs4846886 | 4.24X10^-05^ | 1 | 229154023 | INTRONIC | 0.40 | 0.30 | TTC13 | 0 |
| rs17127984 | 4.30X10^-05^ | 8 | 19116836 | INTERGENIC | 0.05 | 0.02 | SH2D4A | -98647 |
| rs6794192 | 4.37X10^-05^ | 3 | 180510507 | INTERGENIC | 0.26 | 0.17 | ZNF639 | -13738 |
| rs6591222 | 4.57X10^-05^ | 11 | 66307719 | INTRONIC | 0.37 | 0.47 | C11orf80 | 0 |
| rs4953514 | 4.60X10^-05^ | 2 | 47682734 | INTERGENIC | 0.56 | 0.45 | KCNK12 | -31732 |
| rs1901388 | 4.63X10^-05^ | 8 | 39672418 | INTRONIC | 0.27 | 0.19 | ADAM18 | 0 |
| rs6750410 | 4.84X10^-05^ | 2 | 70271234 | INTRONIC | 0.19 | 0.28 | C2orf42 | 0 |
| rs941297 | 4.93X10^-05^ | 7 | 72724982 | DOWNSTREAM | 0.05 | 0.11 | VPS37D | 607 |
| rs1181917 | 4.95X10^-05^ | 7 | 155645607 | INTERGENIC | 0.38 | 0.29 | AF093117.1-1 | 7257 |
| rs10892831 | 5.15X10^-05^ | 11 | 121763566 | INTERGENIC | 0.46 | 0.36 | AP001835.4-1 | -61570 |
| rs6800399 | 5.61X10^-05^ | 3 | 33256297 | INTERGENIC | 0.32 | 0.42 | SUSD5 | -20586 |
| rs6862105 | 5.64X10^-05^ | 5 | 179403248 | INTRONIC | 0.15 | 0.23 | RNF130 | 0 |
| rs743779 | 5.74X10^-05^ | 22 | 35882183 | INTERGENIC | 0.32 | 0.42 | IL2RB | -6207 |
| rs11132917 | 5.94X10^-05^ | 4 | 173000762 | INTRONIC | 0.33 | 0.24 | GALNTL6 | 0 |
| rs10036834 | 6.03X10^-05^ | 5 | 179387034 | INTRONIC | 0.20 | 0.29 | RNF130 | 0 |
| rs17036703 | 6.14X10^-05^ | 2 | 47680334 | INTERGENIC | 0.56 | 0.45 | KCNK12 | -29332 |
| rs2080268 | 6.44X10^-05^ | 12 | 4135535 | INTERGENIC | 0.39 | 0.49 | CCND2 | -117664 |
| rs16910630 | 6.50X10^-05^ | 12 | 15159183 | INTRONIC | 0.24 | 0.34 | RERG | 0 |
| rs9908926 | 6.53X10^-05^ | 17 | 62942012 | INTRONIC | 0.37 | 0.27 | PITPNC1 | 0 |
| rs570111 | 6.55X10^-05^ | 11 | 72895878 | INTRONIC | 0.23 | 0.15 | FAM168A | 0 |
| rs586309 | 6.55X10^-05^ | 11 | 72980761 | INTRONIC | 0.23 | 0.15 | FAM168A | 0 |
| rs4972965 | 6.65X10^-05^ | 2 | 231185659 | INTERGENIC | 0.44 | 0.34 | AC010149.8-3 | 25473 |
| rs7209428 | 6.76X10^-05^ | 17 | 75585536 | INTRONIC | 0.30 | 0.40 | TBC1D16 | 0 |
| rs277067 | 6.79X10^-05^ | 17 | 52920675 | INTRONIC | 0.27 | 0.36 | MSI2 | 0 |
| rs4279641 | 6.81X10^-05^ | 8 | 121353013 | INTRONIC | 0.29 | 0.20 | COL14A1 | 0 |
| rs2112073 | 7.06X10^-05^ | 2 | 50063062 | INTRONIC | 0.46 | 0.36 | NRXN1 | 0 |
| rs11544049 | 7.15X10^-05^ | 7 | 72750277 | 3PRIME_UTR | 0.03 | 0.09 | WBSCR22 | 0 |
| rs34722 | 7.16X10^-05^ | 19 | 35058642 | INTERGENIC | 0.32 | 0.23 | C19orf2 | -47749 |
| rs4739909 | 7.38X10^-05^ | 8 | 83879572 | INTERGENIC | 0.54 | 0.44 | N/A | -9 |
| rs1363025 | 7.55X10^-05^ | 2 | 50068877 | INTRONIC | 0.44 | 0.34 | NRXN1 | 0 |
| rs17258848 | 7.67X10^-05^ | 4 | 127755971 | INTERGENIC | 0.06 | 0.13 | AC097378.2 | 412741 |
| rs1580602 | 7.72X10^-05^ | 16 | 85070375 | INTERGENIC | 0.35 | 0.26 | FOXF1 | -31259 |
| rs1085625 | 7.79X10^-05^ | 3 | 121490740 | INTERGENIC | 0.20 | 0.29 | LRRC58 | 35526 |
| rs984425 | 7.84X10^-05^ | 5 | 9206379 | INTRONIC | 0.29 | 0.21 | SEMA5A | 0 |
| rs6739633 | 8.00X10^-05^ | 2 | 12945998 | INTERGENIC | 0.14 | 0.21 | TRIB2 | 145687 |
| rs2284568 | 8.04X10^-05^ | 21 | 34124461 | INTRONIC | 0.15 | 0.09 | ITSN1 | 0 |
| rs12147331 | 8.18X10^-05^ | 14 | 75027961 | INTERGENIC | 0.29 | 0.20 | JDP2 | 21053 |
| rs9843107 | 8.21X10^-05^ | 3 | 6661203 | INTERGENIC | 0.20 | 0.13 | GRM7 | -216872 |
| rs10171857 | 8.24X10^-05^ | 2 | 28660846 | INTRONIC | 0.21 | 0.14 | PLB1 | 0 |
| rs17096112 | 8.68X10^-05^ | 14 | 71217800 | INTRONIC | 0.32 | 0.42 | SIPA1L1 | 0 |
| rs12731630 | 8.69X10^-05^ | 1 | 222717570 | INTERGENIC | 0.47 | 0.37 | WDR26 | -28946 |
| rs2356007 | 8.82X10^-05^ | 11 | 28708177 | INTERGENIC | 0.39 | 0.49 | METT5D1 | 396547 |
| rs16910980 | 9.06X10^-05^ | 8 | 83873678 | INTERGENIC | 0.54 | 0.44 | N/A | -9 |
| rs1488 | 9.17X10^-05^ | 6 | 161458240 | 3PRIME_UTR | 0.53 | 0.43 | MAP3K4 | 0 |
| rs2042542 | 9.25X10^-05^ | 2 | 218444537 | INTRONIC | 0.30 | 0.39 | TNS1 | 0 |
| rs10904233 | 9.25X10^-05^ | 10 | 4355399 | INTERGENIC | 0.59 | 0.49 | AC022535.6 | -191745 |
| rs9816589 | 9.37X10^-05^ | 3 | 52805587 | SYN_CODING | 0.11 | 0.06 | ITIH3 | 0 |
| rs780222 | 9.50X10^-05^ | 1 | 231727626 | INTERGENIC | 0.43 | 0.33 | KCNK1 | -88747 |
| rs7841995 | 9.54X10^-05^ | 8 | 14819760 | INTRONIC | 0.14 | 0.08 | SGCZ | 0 |
| rs7822661 | 9.75X10^-05^ | 8 | 40139988 | INTERGENIC | 0.26 | 0.35 | C8orf4 | 8010 |
| rs1259321 | 9.87X10^-05^ | 3 | 121691022 | INTERGENIC | 0.31 | 0.22 | FSTL1 | -38489 |
| rs7314281 | 9.88X10^-05^ | 12 | 56320307 | INTERGENIC | 0.06 | 0.02 | B4GALNT1 | -7055 |
