# Supplementary material for: Genomewide Association Study for Determinants of HIV-1 Acquisition and Viral Set Point in HIV-1 Serodiscordant Couples with Quantified Virus Exposure
Source: PLoS One. 2011 Dec 12;6(12):e28632. doi: 10.1371/journal.pone.0028632 (PMC3236203; doi:10.1371/journal.pone.0028632)
Supplement: Table S2 — List of tested variants previously reported to have an association with HIV-1 susceptibility/resistance. SNPs listed are those present on the Illumina HumanHap 1M-Duo (np135) Bead Chips that have been previously implicated in candidate gene studies as having impact on HIV-1 acquisition. Characteristics of the studies that reported those previous associations are described. (DOC) [file pone.0028632.s003.doc]

**Table S2:**

| **Chr** | **Gene** | **SNP** | **Pathway** | **Reported Population(s)** | **Proposed Effect** | **P-value** | **References** |
| --- | --- | --- | --- | --- | --- | --- | --- |
| 3 | CX3CR1 | rs3732378 (T280M) G>A | Chemokine Receptor | Caucasian | recessive homozygosity might increase susceptibility to HIV-1 infection | 0.72 | [1] |
| 3 | CX3CR1 | rs3732379 (V249I) C>T | Chemokine Receptor | Caucasian | recessive homozygosity might increase susceptibility to HIV-1 infection | 0.81 | [1] |
| 17 | CCL2-CCL7-CCL11 | rs1024610 -2136A>T | Chemokines | Caucasian | Decreased susceptibility to HIV-1 infection | 0.84 | [2] |
| 17 | CCL2-CCL7-CCL11 | rs2857657 767C>G | Chemokines | Caucasian | Decreased susceptibility to HIV-1 infection | 0.35 | [2] |
| 17 | CCL2-CCL7-CCL11 | rs4795895 -1385G>A | Chemokines | Caucasian | Decreased susceptibility to HIV-1 infection | 0.62 | [2] |
| 17 | CCL2 | rs1024611 (-2578A>G aka -2518A>G) | Chemokines | Caucasian - not found significant in African American | Reduced Risk of Acquiring HIV-1 | 0.18 | [3] |
| 17 | CCL3 | rs1719134 459C>T | Chemokines | African American | Reduced Risk of Acquiring HIV-1 | 0.74 | [4] |
| 17 | CCL5 | rs2107538 -403G>A | Chemokines | Caucasian | Increase susceptibility | 0.78 | [4-7] |
| 17 | CCL5 | rs2280789 -222T>C | Chemokines | Caucasian and African American | Increased Risk of Acquiring HIV-1 | 0.31 | [8] |
| 10 | CXCL12 | rs1801157 801G>A | Chemokines | Caucasian and African American + South African | Reduced Risk of Acquiring HIV-1 in Caucasians but not African American | 0.29 | [9,10] |
| 1 | IL-10 | rs1800872 -592C>A | Cytokines | Caucasian and African American + African | Increased risk of Acquiring HIV-1 infection | 0.26 | [11,12] |
| 1 | IL-10 | rs1800896 -1082G>A | Cytokines | African | Reduced Risk of Acquiring HIV-1 | 0.45 | [11] |
| 11 | IL-18 | rs1946518 -607A>C | Cytokines | Brazilian | Increased risk of Acquiring HIV-1 infection (Paediatric) | 0.50 | [13] |
| 5 | IRF1 | rs17848424 aka rs2070729 6516G>T | Intracellular signaling | Kenya | Resistance to HIV-1 infection | 0.01 | [14] |
| 11 | TRIM5 | rs3740996 127C>T (H43Y) | Viral restriction factor | Caucasian and African American | Reduced Risk of Acquiring HIV-1 in African American | 0.956 | [15] |
| 11 | TRIM5 | rs10838525 407G>A (R136Q) | Viral restriction factor | Caucasian and African American | Reduced Risk of Acquiring HIV-1 in African American | 0.78 | [15] |
| 10 | MBL2 | rs5030737 (R52C) | Acute phase reactant | Caucasian | Increased risk of Acquiring HIV-1 infection | 0.47 | [16-20] |
| 10 | MBL2 | rs1800450 (G54D) | Acute phase reactant | Caucasian | Increased risk of Acquiring HIV-1 infection | 0.79 | [16-20] |
| 10 | MBL2 | rs1800451 (G57E) | Acute phase reactant | Caucasian + Gabonese | Increased risk of Acquiring HIV-1 infection | 0.02 | [16-21] |
| 7 | ABCB1 | rs1045642 3435C>T | Other | Caucasian | Reduced Risk of Acquiring HIV-1 | 0.66 | [22] |
| 12 | CD4 | rs28919570 868C>T | HIV co-receptor | Kenya | Increased risk of Acquiring HIV-1 infection | 0.55 | [23,24] |

References for Table S2

1. Faure S, Meyer L, Costagliola D, Vaneensberghe C, Genin E, et al. (2000) Rapid progression to AIDS in HIV+ individuals with a structural variant of the chemokine receptor CX3CR1. Science 287: 2274-2277.

2. Modi WS, Goedert JJ, Strathdee S, Buchbinder S, Detels R, et al. (2003) MCP-1-MCP-3-Eotaxin gene cluster influences HIV-1 transmission. AIDS 17: 2357-2365.

3. Gonzalez E, Rovin BH, Sen L, Cooke G, Dhanda R, et al. (2002) HIV-1 infection and AIDS dementia are influenced by a mutant MCP-1 allele linked to increased monocyte infiltration of tissues and MCP-1 levels. Proc Natl Acad Sci U S A 99: 13795-13800.

4. Gonzalez E, Dhanda R, Bamshad M, Mummidi S, Geevarghese R, et al. (2001) Global survey of genetic variation in CCR5, RANTES, and MIP-1alpha: impact on the epidemiology of the HIV-1 pandemic. Proc Natl Acad Sci U S A 98: 5199-5204.

5. Fernandez RM, Borrego S, Marcos I, Rubio A, Lissen E, et al. (2003) Fluorescence resonance energy transfer analysis of the RANTES polymorphisms -403G --> A and -28G --> C: evaluation of both variants as susceptibility factors to HIV type 1 infection in the Spanish population. AIDS Res Hum Retroviruses 19: 349-352.

6. Liu H, Hwangbo Y, Holte S, Lee J, Wang C, et al. (2004) Analysis of genetic polymorphisms in CCR5, CCR2, stromal cell-derived factor-1, RANTES, and dendritic cell-specific intercellular adhesion molecule-3-grabbing nonintegrin in seronegative individuals repeatedly exposed to HIV-1. J Infect Dis 190: 1055-1058.

7. McDermott DH, Colla JS, Kleeberger CA, Plankey M, Rosenberg PS, et al. (2000) Genetic polymorphism in CX3CR1 and risk of HIV disease. Science 290: 2031.

8. An P, Nelson GW, Wang L, Donfield S, Goedert JJ, et al. (2002) Modulating influence on HIV/AIDS by interacting RANTES gene variants. Proc Natl Acad Sci U S A 99: 10002-10007.

9. Modi WS, Scott K, Goedert JJ, Vlahov D, Buchbinder S, et al. (2005) Haplotype analysis of the SDF-1 (CXCL12) gene in a longitudinal HIV-1/AIDS cohort study. Genes Immun 6: 691-698.

10. Petersen DC, Glashoff RH, Shrestha S, Bergeron J, Laten A, et al. (2005) Risk for HIV-1 infection associated with a common CXCL12 (SDF1) polymorphism and CXCR4 variation in an African population. J Acquir Immune Defic Syndr 40: 521-526.

11. Naicker DD, Werner L, Kormuth E, Passmore JA, Mlisana K, et al. (2009) Interleukin-10 promoter polymorphisms influence HIV-1 susceptibility and primary HIV-1 pathogenesis. J Infect Dis 200: 448-452.

12. Shin HD, Winkler C, Stephens JC, Bream J, Young H, et al. (2000) Genetic restriction of HIV-1 pathogenesis to AIDS by promoter alleles of IL10. Proc Natl Acad Sci U S A 97: 14467-14472.

13. Segat L, Bevilacqua D, Boniotto M, Arraes LC, de Souza PR, et al. (2006) IL-18 gene promoter polymorphism is involved in HIV-1 infection in a Brazilian pediatric population. Immunogenetics 58: 471-473.

14. Ball TB, Ji H, Kimani J, McLaren P, Marlin C, et al. (2007) Polymorphisms in IRF-1 associated with resistance to HIV-1 infection in highly exposed uninfected Kenyan sex workers. AIDS 21: 1091-1101.

15. Javanbakht H, An P, Gold B, Petersen DC, O'Huigin C, et al. (2006) Effects of human TRIM5alpha polymorphisms on antiretroviral function and susceptibility to human immunodeficiency virus infection. Virology.

16. Boniotto M, Braida L, Pirulli D, Arraes L, Amoroso A, et al. (2003) MBL2 polymorphisms are involved in HIV-1 infection in Brazilian perinatally infected children. AIDS 17: 779-780.

17. Garred P, Madsen HO, Balslev U, Hofmann B, Pedersen C, et al. (1997) Susceptibility to HIV infection and progression of AIDS in relation to variant alleles of mannose-binding lectin. Lancet 349: 236-240.

18. Malik S, Arias M, Di Flumeri C, Garcia LF, Schurr E (2003) Absence of association between mannose-binding lectin gene polymorphisms and HIV-1 infection in a Colombian population. Immunogenetics 55: 49-52.

19. Pastinen T, Liitsola K, Niini P, Salminen M, Syvanen AC (1998) Contribution of the CCR5 and MBL genes to susceptibility to HIV type 1 infection in the Finnish population. AIDS Res Hum Retroviruses 14: 695-698.

20. Vallinoto AC, Menezes-Costa MR, Alves AE, Machado LF, de Azevedo VN, et al. (2006) Mannose-binding lectin gene polymorphism and its impact on human immunodeficiency virus 1 infection. Mol Immunol 43: 1358-1362.

21. Mombo LE, Lu CY, Ossari S, Bedjabaga I, Sica L, et al. (2003) Mannose-binding lectin alleles in sub-Saharan Africans and relation with susceptibility to infections. Genes Immun 4: 362-367.

22. Fellay J, Marzolini C, Meaden ER, Back DJ, Buclin T, et al. (2002) Response to antiretroviral treatment in HIV-1-infected individuals with allelic variants of the multidrug resistance transporter 1: a pharmacogenetics study. Lancet 359: 30-36.

23. Choi RY, Farquhar C, Juno J, Mbori-Ngacha D, Lohman-Payne B, et al. (2010) Infant CD4 C868T polymorphism is associated with increased human immunodeficiency virus (HIV-1) acquisition. Clin Exp Immunol 160: 461-465.

24. Oyugi JO, Vouriot FC, Alimonti J, Wayne S, Luo M, et al. (2009) A common CD4 gene variant is associated with an increased risk of HIV-1 infection in Kenyan female commercial sex workers. J Infect Dis 199: 1327-1334.
