# Supplementary material for: Genomewide Association Study for Determinants of HIV-1 Acquisition and Viral Set Point in HIV-1 Serodiscordant Couples with Quantified Virus Exposure
Source: PLoS One. 2011 Dec 12;6(12):e28632. doi: 10.1371/journal.pone.0028632 (PMC3236203; doi:10.1371/journal.pone.0028632)
Supplement: Table S3 — Variants with p<10−5 in HIV-1 set point analysis. SNP rs identifier, uncorrected p-value, chromosome number and basepair position (build 36.3, hg18), and name and distance to closest gene are as indicated. (DOC) [file pone.0028632.s004.doc]

**Table S3:**

| **SNP** | **P** | **Chromosome** | **Coordinate** | **SNP type** | **Closest gene** | **Distance to Gene** |
| --- | --- | --- | --- | --- | --- | --- |
| rs13111989 | 2.15X10^-07^ | 4 | 177779967 | INTERGENIC | VEGFC | 61718 |
| rs10484434 | 1.14X10^-06^ | 6 | 26139592 | INTRONIC | HIST1H4A | 0 |
| rs4404602 | 1.20X10^-06^ | 4 | 66153408 | INTRONIC | EPHA5 | 0 |
| rs11755492 | 1.75X10^-06^ | 6 | 26154137 | 3PRIME_UTR | HIST1H3C | 0 |
| rs10455590 | 2.04X10^-06^ | 6 | 66654915 | INTERGENIC | AL391500.13 | -50247 |
| rs5746647 | 2.26X10^-06^ | 22 | 15437138 | DOWNSTREAM | KB-67B5.12 | 1611 |
| rs6776297 | 4.60X10^-06^ | 3 | 32353275 | INTRONIC | CMTM8 | 0 |
| rs7674482 | 4.79X10^-06^ | 4 | 45562218 | INTERGENIC | AC095058.3 | -129675 |
| rs8061903 | 5.02X10^-06^ | 16 | 9289830 | INTERGENIC | AC087190.5-2 | -131591 |
| rs12644436 | 5.51X10^-06^ | 4 | 89024232 | INTERGENIC | HSP90AB3P | -7787 |
| rs17689437 | 5.54X10^-06^ | 16 | 67157852 | 3PRIME_UTR | ZFP90 | 0 |
| rs1493682 | 5.63X10^-06^ | 4 | 121260734 | INTERGENIC | MAD2L1 | -53323 |
| rs249292 | 6.35X10^-06^ | 16 | 9320305 | INTERGENIC | AC087190.5-2 | -162066 |
| rs4601292 | 6.37X10^-06^ | 8 | 67014285 | INTERGENIC | DNAJC5B | -82060 |
| rs264943 | 6.85X10^-06^ | 2 | 103664973 | INTERGENIC | N/A | -9 |
| rs1938105 | 8.84X10^-06^ | 6 | 66567647 | INTERGENIC | RP11-707M13.1 | 11746 |
| rs1000916 | 9.13X10^-06^ | 2 | 31168059 | INTRONIC | GALNT14 | 0 |
| rs1381964 | 9.43X10^-06^ | 4 | 89022387 | INTERGENIC | HSP90AB3P | -9632 |
| rs6855605 | 9.50X10^-06^ | 4 | 66138687 | INTRONIC | EPHA5 | 0 |
| rs1588127 | 1.17X10^-05^ | 15 | 47635303 | INTRONIC | C15orf33 | 0 |
| rs10519233 | 1.17X10^-05^ | 15 | 47693662 | INTRONIC | C15orf33 | 0 |
| rs11721867 | 1.28X10^-05^ | 4 | 45553221 | INTERGENIC | AC095058.3 | -138672 |
| rs1710563 | 1.33X10^-05^ | 10 | 126118542 | INTERGENIC | NKX1-2 | 7451 |
| rs4690458 | 1.44X10^-05^ | 4 | 177793749 | INTERGENIC | VEGFC | 47936 |
| rs6494221 | 1.50X10^-05^ | 15 | 58803399 | INTRONIC | RORA | 0 |
| rs13075697 | 1.51X10^-05^ | 3 | 28687243 | INTERGENIC | C3orf53 | -94421 |
| rs9882449 | 1.56X10^-05^ | 3 | 157320075 | UPSTREAM | KCNAB1 | -1020 |
| rs11124555 | 2.01X10^-05^ | 2 | 37029717 | INTRONIC | STRN | 0 |
| rs17596719 | 2.16X10^-05^ | 6 | 26205173 | INTRONIC | HIST1H4A | 0 |
| rs6549869 | 2.49X10^-05^ | 3 | 28676500 | INTERGENIC | C3orf53 | -83678 |
| rs4561706 | 2.51X10^-05^ | 2 | 31170013 | INTRONIC | GALNT14 | 0 |
| rs11945046 | 2.64X10^-05^ | 4 | 94814573 | INTRONIC | GRID2 | 0 |
| rs11946557 | 2.64X10^-05^ | 4 | 94816124 | INTRONIC | GRID2 | 0 |
| rs16958854 | 2.68X10^-05^ | 13 | 87205851 | INTERGENIC | SLITRK5 | 75982 |
| rs1565106 | 2.87X10^-05^ | 4 | 188373301 | INTERGENIC | AC097521.2-1 | 420148 |
| rs4441811 | 3.02X10^-05^ | 4 | 66204084 | INTRONIC | EPHA5 | 0 |
| rs7082385 | 3.04X10^-05^ | 10 | 19901296 | DOWNSTREAM | C10orf112 | 4788 |
| rs2611339 | 3.10X10^-05^ | 8 | 61220386 | INTERGENIC | CA8 | 43591 |
| rs992874 | 3.18X10^-05^ | 2 | 53385960 | INTERGENIC | AC069157.8 | 165129 |
| rs40511 | 3.27X10^-05^ | 16 | 9313335 | INTERGENIC | AC087190.5-2 | -155096 |
| rs12195428 | 3.34X10^-05^ | 6 | 22615964 | INTERGENIC | HDGFL1 | -61693 |
| rs6994725 | 3.46X10^-05^ | 8 | 67007297 | INTERGENIC | DNAJC5B | -89048 |
| rs12526082 | 3.46X10^-05^ | 6 | 17438834 | INTERGENIC | RBM24 | 36758 |
| rs6926482 | 3.62X10^-05^ | 6 | 54809644 | INTERGENIC | FAM83B | -9884 |
| rs31313 | 3.69X10^-05^ | 5 | 16743754 | INTRONIC | MYO10 | 0 |
| rs3788412 | 3.77X10^-05^ | 22 | 28080186 | INTRONIC | AP1B1 | 0 |
| rs16938058 | 3.85X10^-05^ | 9 | 20401354 | INTRONIC | MLLT3 | 0 |
| rs6531719 | 3.93X10^-05^ | 4 | 39163696 | INTERGENIC | AC021148.8 | 5083 |
| rs4741881 | 3.94X10^-05^ | 9 | 4039110 | INTRONIC | GLIS3 | 0 |
| rs4611973 | 3.94X10^-05^ | 4 | 66198991 | INTRONIC | EPHA5 | 0 |
| rs12529685 | 4.07X10^-05^ | 6 | 66601369 | DOWNSTREAM | AL391500.13 | 2196 |
| rs6472235 | 4.29X10^-05^ | 8 | 66984584 | INTERGENIC | PDE7A | -67731 |
| rs6472236 | 4.29X10^-05^ | 8 | 66995235 | INTERGENIC | PDE7A | -78382 |
| rs2681543 | 4.30X10^-05^ | 8 | 61219047 | INTERGENIC | CA8 | 44930 |
| rs13286283 | 4.34X10^-05^ | 9 | 25019338 | INTERGENIC | AL627260.7 | 59488 |
| rs5986904 | 4.49X10^-05^ | X | 153961021 | INTRONIC | BRCC3 | 0 |
| rs3740898 | 4.52X10^-05^ | 11 | 124781067 | INTRONIC | PKNOX2 | 0 |
| rs7243538 | 4.54X10^-05^ | 18 | 53713462 | INTERGENIC | AC022724.8-1 | 56268 |
| rs1547458 | 4.62X10^-05^ | 3 | 72211574 | INTERGENIC | AC105265.4 | -7206 |
| rs9491205 | 4.63X10^-05^ | 6 | 124997216 | INTRONIC | NKAIN2;TCBA1 | 0 |
| rs2216831 | 4.66X10^-05^ | 2 | 31172342 | INTRONIC | GALNT14 | 0 |
| rs12809335 | 4.76X10^-05^ | 12 | 30739942 | 5PRIME_UTR | IPO8 | 0 |
| rs1362166 | 4.86X10^-05^ | 7 | 34618870 | INTRONIC | AC005582.1 | 0 |
| rs12661820 | 5.08X10^-05^ | 6 | 166904646 | INTRONIC | RPS6KA2 | 0 |
| rs2162948 | 5.41X10^-05^ | 16 | 9316811 | INTERGENIC | AC087190.5-2 | -158572 |
| rs2771122 | 5.62X10^-05^ | 1 | 151579268 | INTRONIC | PGLYRP4 | 0 |
| rs3113749 | 5.73X10^-05^ | 4 | 181729671 | DOWNSTREAM | AC096590.4 | 1940 |
| rs6998258 | 5.94X10^-05^ | 8 | 67201943 | 5PRIME_UTR | TRIM55 | 0 |
| rs17482833 | 5.98X10^-05^ | 15 | 47758787 | INTERGENIC | DTWD1 | 35636 |
| rs11927605 | 6.02X10^-05^ | 3 | 157313272 | INTERGENIC | KCNAB1 | -7823 |
| rs2204792 | 6.14X10^-05^ | 1 | 169253902 | DOWNSTREAM | C1orf129 | 3877 |
| rs9389549 | 6.15X10^-05^ | 6 | 138384712 | INTERGENIC | RP11-240M16.2 | 25735 |
| rs4674616 | 6.32X10^-05^ | 2 | 222426022 | INTERGENIC | AC079834.8-1 | 260231 |
| rs11063009 | 6.42X10^-05^ | 12 | 4109753 | INTERGENIC | CCND2 | -143446 |
| rs7041793 | 6.48X10^-05^ | 9 | 20418137 | INTRONIC | MLLT3 | 0 |
| rs10834970 | 6.63X10^-05^ | 11 | 26336745 | INTRONIC | ANO3 | 0 |
| rs16561 | 6.68X10^-05^ | 17 | 28389865 | INTRONIC | ACCN1 | 0 |
| rs6590386 | 6.68X10^-05^ | 11 | 128814634 | INTRONIC | BARX2 | 0 |
| rs8064314 | 6.69X10^-05^ | 17 | 32932505 | INTRONIC | DUSP14 | 0 |
| rs3818198 | 7.02X10^-05^ | 20 | 50210433 | INTRONIC | ZFP64 | 0 |
| rs17134126 | 7.03X10^-05^ | 7 | 51052050 | 3PRIME_UTR | COBL | 0 |
| rs4422467 | 7.32X10^-05^ | 4 | 66177240 | INTRONIC | EPHA5 | 0 |
| rs5935919 | 7.39X10^-05^ | X | 15199974 | UPSTREAM | ASB9 | -1870 |
| rs450788 | 7.39X10^-05^ | 4 | 66806817 | INTERGENIC | MIRN1269 | -18320 |
| rs2053256 | 7.43X10^-05^ | 17 | 4055397 | INTRONIC | ANKFY1 | 0 |
| rs1534608 | 7.55X10^-05^ | 2 | 22756991 | INTERGENIC | AC104807.5-2 | -180938 |
| rs16892299 | 7.72X10^-05^ | 5 | 23297599 | INTERGENIC | AC010460.7 | -37422 |
| rs9905713 | 7.84X10^-05^ | 17 | 52656368 | DOWNSTREAM | AC003950.1 | 4812 |
| rs7126004 | 8.03X10^-05^ | 11 | 128813952 | INTRONIC | BARX2 | 0 |
| rs17074536 | 8.22X10^-05^ | 4 | 184417378 | INTRONIC | WWC2 | 0 |
| rs12050270 | 8.40X10^-05^ | 14 | 25909266 | INTERGENIC | NOVA1 | 75663 |
| rs9292982 | 8.43X10^-05^ | 5 | 21540338 | INTERGENIC | AC138951.2-3 | 6296 |
| rs6024437 | 8.51X10^-05^ | 20 | 53813962 | INTERGENIC | CBLN4 | 191942 |
| rs10744716 | 8.57X10^-05^ | 12 | 6810111 | NON_SYN_CODING | LEPREL2 | 0 |
| rs2162652 | 8.68X10^-05^ | 3 | 141651667 | INTRONIC | CLSTN2 | 0 |
| rs11789634 | 8.84X10^-05^ | 9 | 103713366 | INTERGENIC | AL133413.5 | -117439 |
| rs4853422 | 8.85X10^-05^ | 2 | 78970230 | INTERGENIC | AC069162.8-1 | 6862 |
| rs16860968 | 8.88X10^-05^ | 4 | 47815628 | INTRONIC | TXK | 0 |
| rs4415013 | 8.99X10^-05^ | 4 | 135034486 | INTERGENIC | AC133605.3 | 104580 |
| rs6024460 | 9.03X10^-05^ | 20 | 53828948 | INTERGENIC | CBLN4 | 176956 |
| rs10827678 | 9.06X10^-05^ | 10 | 36960327 | INTERGENIC | AL354660.9 | -5255 |
| rs2102360 | 9.12X10^-05^ | 8 | 41807985 | INTRONIC | ANK1 | 0 |
| rs16874223 | 9.20X10^-05^ | 6 | 13670256 | INTERGENIC | SIRT5 | -12556 |
| rs6912037 | 9.21X10^-05^ | 6 | 75247853 | INTERGENIC | RP11-554D15.2 | 129032 |
| rs6693831 | 9.44X10^-05^ | 1 | 67493455 | INTRONIC | IL23R | 0 |
| rs5999230 | 9.52X10^-05^ | 22 | 32887399 | INTERGENIC | LARGE | -240989 |
| rs10508819 | 9.69X10^-05^ | 10 | 36119299 | INTERGENIC | RP11-382K22.1 | 92328 |
| rs6538249 | 9.75X10^-05^ | 12 | 89785736 | INTERGENIC | C12orf37 | -70828 |
| rs4727329 | 9.77X10^-05^ | 7 | 95360863 | INTRONIC | DYNC1I1 | 0 |
